# Elevated atmospheric CO_2_ alters the microbial community composition and metabolic potential to mineralize organic phosphorus in the rhizosphere of wheat

**DOI:** 10.1186/s40168-021-01203-w

**Published:** 2022-01-24

**Authors:** Jian Jin, Christian Krohn, Ashley E. Franks, Xiaojuan Wang, Jennifer L. Wood, Steve Petrovski, Malcolm McCaskill, Steven Batinovic, Zhihuang Xie, Caixian Tang

**Affiliations:** 1 Department of Animal, Plant and Soil Sciences, Centre for AgriBioscience, La Trobe University, Melbourne Campus, Bundoora, Victoria 3086 Australia; 2 Department of Physiology, Anatomy and Microbiology, La Trobe University, Melbourne Campus, Bundoora, Victoria 3086 Australia; 3 Centre for Future Landscapes, La Trobe University, Melbourne Campus, Bundoora, Victoria 3086 Australia; 4 Agriculture Victoria Research, Department of Jobs, Precincts and Regions, Victoria 3300 Hamilton, Australia; 5 Key Laboratory of Mollisols Agroecology, Northeast Institute of Geography and Agroecology, Chinese Academy of Sciences, Harbin, 150081 China

**Keywords:** Climate change, Phytate mineralization, Microbial phosphorus, Microbial phylotypes, Metabolic pathway, Rhizobox

## Abstract

**Background:**

Understanding how elevated atmospheric CO_2_ (eCO_2_) impacts on phosphorus (P) transformation in plant rhizosphere is critical for maintaining ecological sustainability in response to climate change, especially in agricultural systems where soil P availability is low.

**Methods:**

This study used rhizoboxes to physically separate rhizosphere regions (plant root-soil interface) into 1.5-mm segments. Wheat plants were grown in rhizoboxes under eCO_2_ (800 ppm) and ambient CO_2_ (400 ppm) in two farming soils, Chromosol and Vertosol, supplemented with phytate (organic P). Photosynthetic carbon flow in the plant-soil continuum was traced with ^13^CO_2_ labeling. Amplicon sequencing was performed on the rhizosphere-associated microbial community in the root-growth zone, and 1.5 mm and 3 mm away from the root.

**Results:**

Elevated CO_2_ accelerated the mineralization of phytate in the rhizosphere zones, which corresponded with increases in plant-derived ^13^C enrichment and the relative abundances of discreet phylogenetic clades containing Bacteroidetes and Gemmatimonadetes in the bacterial community, and *Funneliformis* affiliated to arbuscular mycorrhizas in the fungal community. Although the amplicon sequence variants (ASVs) associated the stimulation of phytate mineralization under eCO_2_ differed between the two soils, these ASVs belonged to the same phyla associated with phytase and phosphatase production. The symbiotic mycorrhizas in the rhizosphere of wheat under eCO_2_ benefited from increased plant C supply and increased P access from soil. Further supportive evidence was the eCO_2_-induced increase in the genetic pool expressing the pentose phosphate pathway, which is the central pathway for biosynthesis of RNA/DNA precursors.

**Conclusions:**

The results suggested that an increased belowground carbon flow under eCO_2_ stimulated bacterial growth, changing community composition in favor of phylotypes capable of degrading aromatic P compounds. It is proposed that energy investments by bacteria into anabolic processes increase under eCO_2_ to level microbial P-use efficiencies and that synergies with symbiotic mycorrhizas further enhance the competition for and mineralization of organic P.

Video Abstract

**Supplementary Information:**

The online version contains supplementary material available at 10.1186/s40168-021-01203-w.

## Introduction

Phosphorus (P) is fundamentally important to soil biota as a major building block of life [1, 2]. Although many soils have extensive stocks of total P, orthophosphate, low in many soils, is the only form of P available to plants and microbes [3]. Non-labile P in soils requires mobilization to increase P availability and improve P nutrition of plants and other biota [4, 5]. Since organic P comprises up to 80% of total P in soils [6, 7], the mineralization of organic P by soil microorganisms could have the potential to be a prominent process in P transformation, especially the mineralization of a dominant component of organic P, such as phytate accounting for 50–60% of the organic P.

Climate change has the potential to impact organic P transformation. One of most important climate change factors, elevated atmospheric CO_2_ concentration (eCO_2_), would considerably accelerate the mineralization of soil organic P [8, 9]. The acceleration of organic P mineralization is attributed to modified plant-soil-microbe interactions due to changes in the plant carbon (C) flow belowground [10–12]. Elevated CO_2_ is known to enhance microbial growth and activity in the rhizosphere of many plant species, as a consequence of plant C efflux in the forms of root exudates and mucilages. Synchronously, plant P demand increases under eCO_2_ as well [10]. The competition for P between plants and soil microbes in P-limited soils, and balancing C/P stoichiometry in microorganisms with increasing P-use efficiency for microbial population growth likely intensify the mobilization of soil organic P [10–13]. In a study where crops were grown under eCO_2_ (550 ppm) over a 7-year period, Jin et al. [11] reported a significant decrease in organic P concentration in two soils, demonstrating the microbial contribution to organic P mineralization.

However, the microbial mechanisms on the acceleration of soil organic P mineralization under eCO_2_ remain largely unknown. While plant roots release extracellular phosphatases, much of this enzyme activity is restricted to the root surface [14–16]. Thus, active mineralization of soil organic P mainly takes place where soil microorganisms interact with plant-derived C. The phylogenetic structure of soil microbial communities has been found to be altered by eCO_2_ in a number of natural and agricultural systems [17–19], which would be associated with the alternation of metabolic potentials in terms of recalcitrant carbon degradation and nutrient mineralization [11, 20]. Recent studies revealed that Actinomycetales, Rhizobiales, Acidobacteriales, and Solibacterales were the potential drivers of P turnover in a beech (*Fagus sylvatica*)-dominated forest soil [5], and *Advenella* and *Cellulosimicrobium* were identified as phytase-producing bacteria to mineralize phytate in soils [21]. Furthermore, in a fungal community, numerous mycorrhizal fungi are able to hydrolyse organic P [22]. Nevertheless, knowledge about the mineralization of organic P under eCO_2_ is mostly restricted to what specific microbial phylotypes contribute to the mineralization of major organic P compounds in the rhizosphere of crops grown under eCO_2_ and whether those phylotypes that enhance P turnover are universal across different soils. Relevant studies help us to better understand the impacts of climate change on the microbial ecological service on P availability to crops grown in P-deficient soils, given that approximately 5.7 billion ha of arable lands around the world are P-deficient and the natural P reserves for fertilizers are diminishing [23, 24].

This study quantified the microbial contribution to mineralization of organic P under eCO_2_ by exploring the community-wide genetic profiling of soil microorganisms, including bacterial and fungal communities and their functions in relation to mineralization of a major organic P compound, phytate, in the rhizosphere of wheat. We hypothesized that (1) eCO_2_ would increase mineralization of organic P compounds in the rhizosphere, resulting from changes in relevant microbial phylotypes and metabolic potentials of soil microbial communities; (2) independent of soil type, eCO_2_ would intensify microbial P use in P-deficient soils to such an extent that microbial phylogeny and functional potentials are significantly altered in the process.

## Results

### Photosynthetic C allocation and phytate mineralization under elevated CO_2_ (eCO_2_)

Elevated CO_2_ significantly increased total biomass of wheat and plant P contents in both Chromosol and Vertosol (Fig. S1). Furthermore, eCO_2_ resulted in increased C fixation by wheat plants which was indicated by increased ^13^C enrichment in the shoot and root by 32% and 50%, respectively, across both soils.

**Fig. 1:**
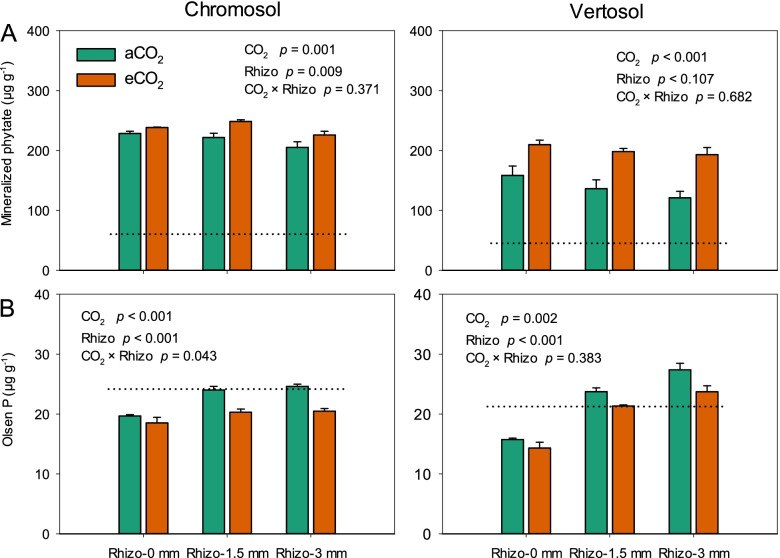
The mineralized phytate (**A**) and Olsen P (**B**) in the rhizosphere compartments: 0, 1.5, and 3 mm away from the root growth zone of wheat grown for 10 weeks in Chromosol and Vertosol under elevated CO_2_ (eCO_2_, 800 ppm) and ambient CO_2_ (aCO_2_, 400 ppm). Error bars are standard error (*n*=6). The dotted lines represent the corresponding value in the bulk soil. The statistical significance for the main effects of CO_2_ and rhizosphere (Rhizo) and their interaction are presented

Elevated CO_2_ further increased C deposition belowground as shown by a 1.6-fold of increase in ^13^C enrichment in the rhizosphere soil across the Chromosol and Vertosol (Fig. S1). Furthermore, eCO_2_ significantly increased the amount of mineralized phytate in the rhizosphere of wheat (9% and 45% increase in the Chromosol and Vertosol, respectively) (Fig. 1A). By contrast, eCO_2_ decreased the concentration of Olsen P by 11% and 13% across the rhizosphere compartments for the Chromosol and Vertosol, respectively (Fig. 1B).

### Microbial C and P, and respiration in relation to phytate mineralization

**Fig. 2:**
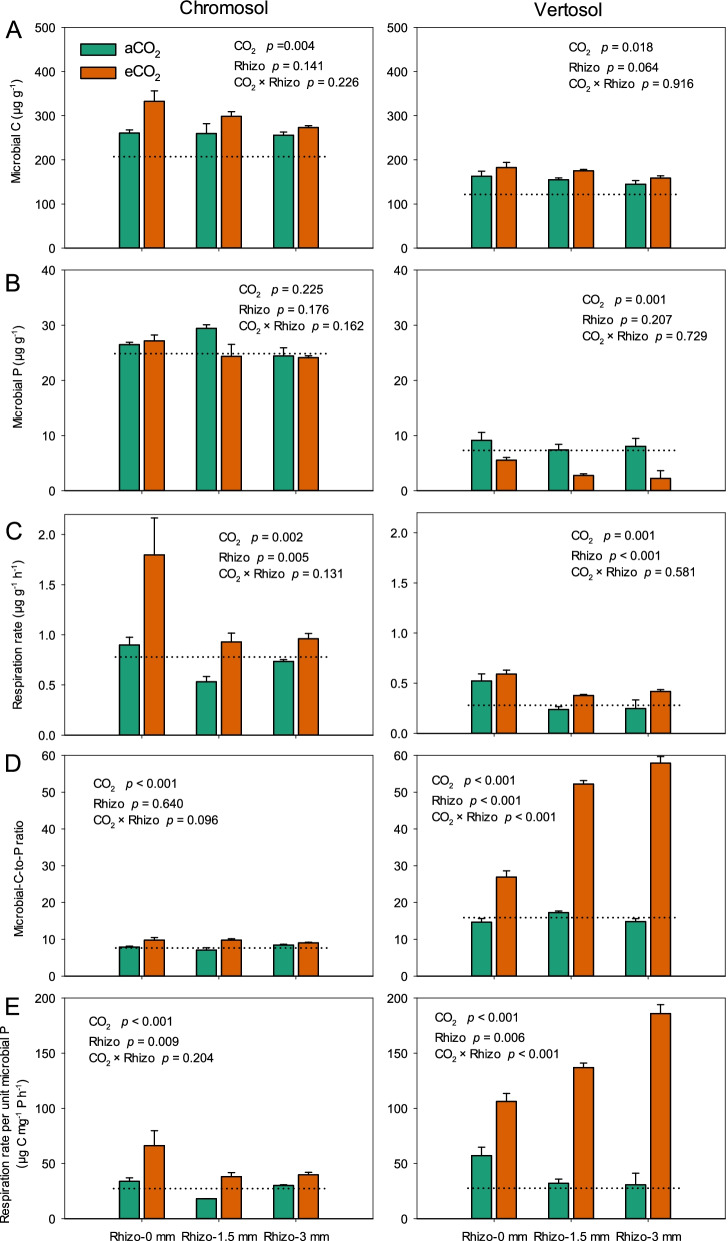
The microbial biomass C (**A**), microbial P (**B**), soil respiration rate (**C**), microbial-C-to-P ratio (**D**), and respiration rate per unit microbial P (**E**) in the rhizosphere compartments: 0, 1.5, and 3 mm away from the root growth zone of wheat grown in Chromosol and Vertosol for 10 weeks under elevated CO_2_ (eCO_2_, 800 ppm) and ambient CO_2_ (aCO_2_, 400 ppm). Error bars are standard error (SE, *n*=6). The dotted lines represent the corresponding values in the bulk soil. The statistical significance for the main effects of CO_2_ and rhizosphere (Rhizo) and their interaction are presented

**Fig. 3:**
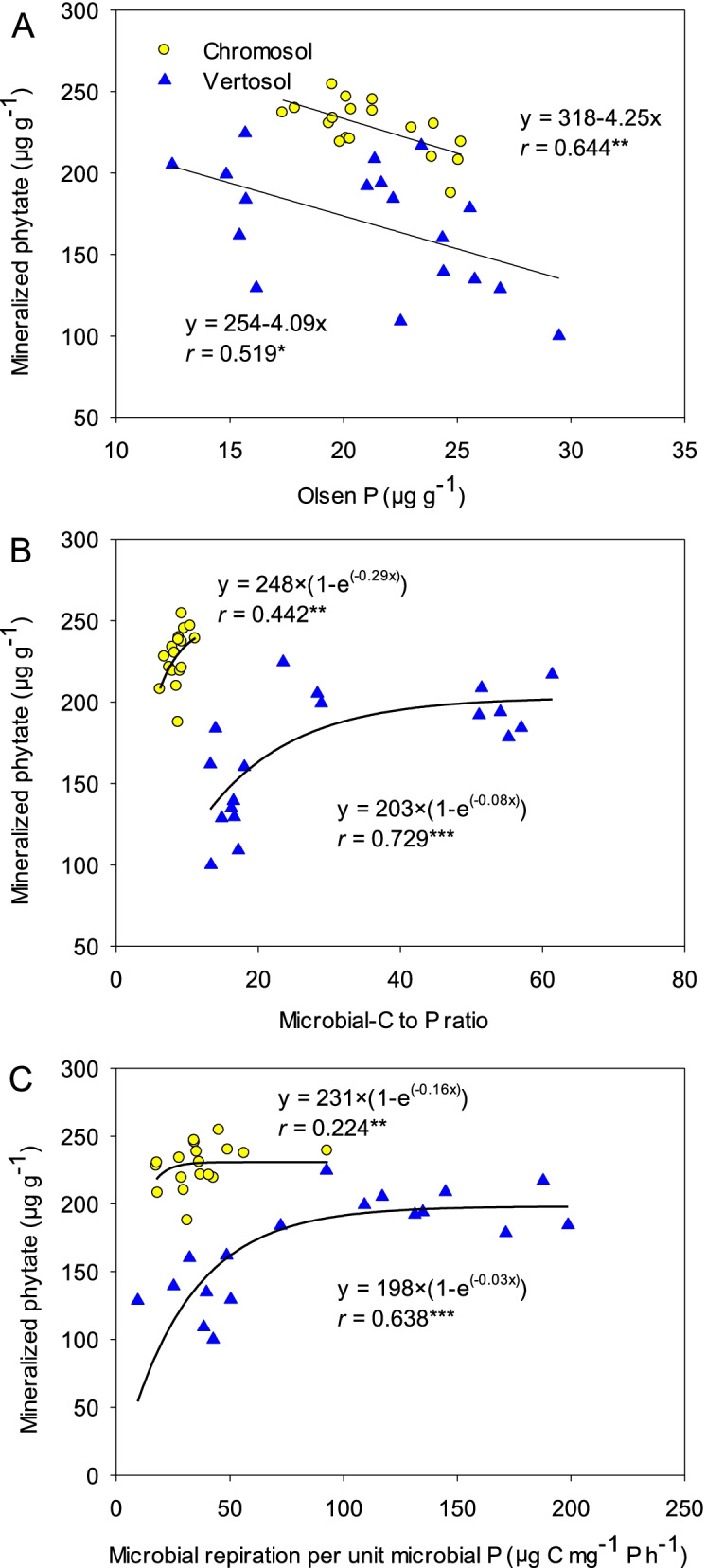
The relationships between mineralized phytate and Olsen P (**A**), microbial-C-to-P ratio (**B**), and microbial respiration per unit microbial P (**C**) in the rhizosphere of wheat plants grown for 10 weeks in Chromosol and Vertosol under elevated CO_2_ (eCO_2_, 800 ppm) and ambient CO_2_ (aCO_2_, 400 ppm). *, **, and *** indicate significance at *p* < 0.05, *p* < 0.01, and *p* < 0.001, respectively

Elevated CO_2_ altered the microbiological characteristics in the rhizosphere. Microbial biomass C increased from 258 μg g^-1^ (±12.2 SE) under aCO_2_ to 301 μg g^-1^ (±13.1 SE) under eCO_2_ across the rhizosphere compartments of wheat grown in the Chromosol. In the Vertosol, eCO_2_ increased microbial biomass C from 152 (±7.9 SE) to 172 μg g^-1^ (±6.6 SE) (Fig. 2A). However, eCO_2_ did not increase microbial P in the Chromosol decreased it in the rhizosphere in the Vertosol (Fig. 2B). The microbial respiration rate in the rhizosphere significantly (*p* < 0.01) increased under eCO_2_ in both soils (Fig. 2C). With the considerable increases in microbial biomass C, eCO_2_ dramatically increased the microbial-C-to-P ratio and microbial respiration rate per unit microbial P in both soils (Fig. 2D, E). The mineralized P was correlated negatively with Olsen P but positively with the microbial-C-to-P ratio and microbial respiration rate per unit microbial P in two soils (Fig. 3).

### Bacterial and fungal diversity under eCO_2_ and their association with phytate mineralization

**Fig. 4:**
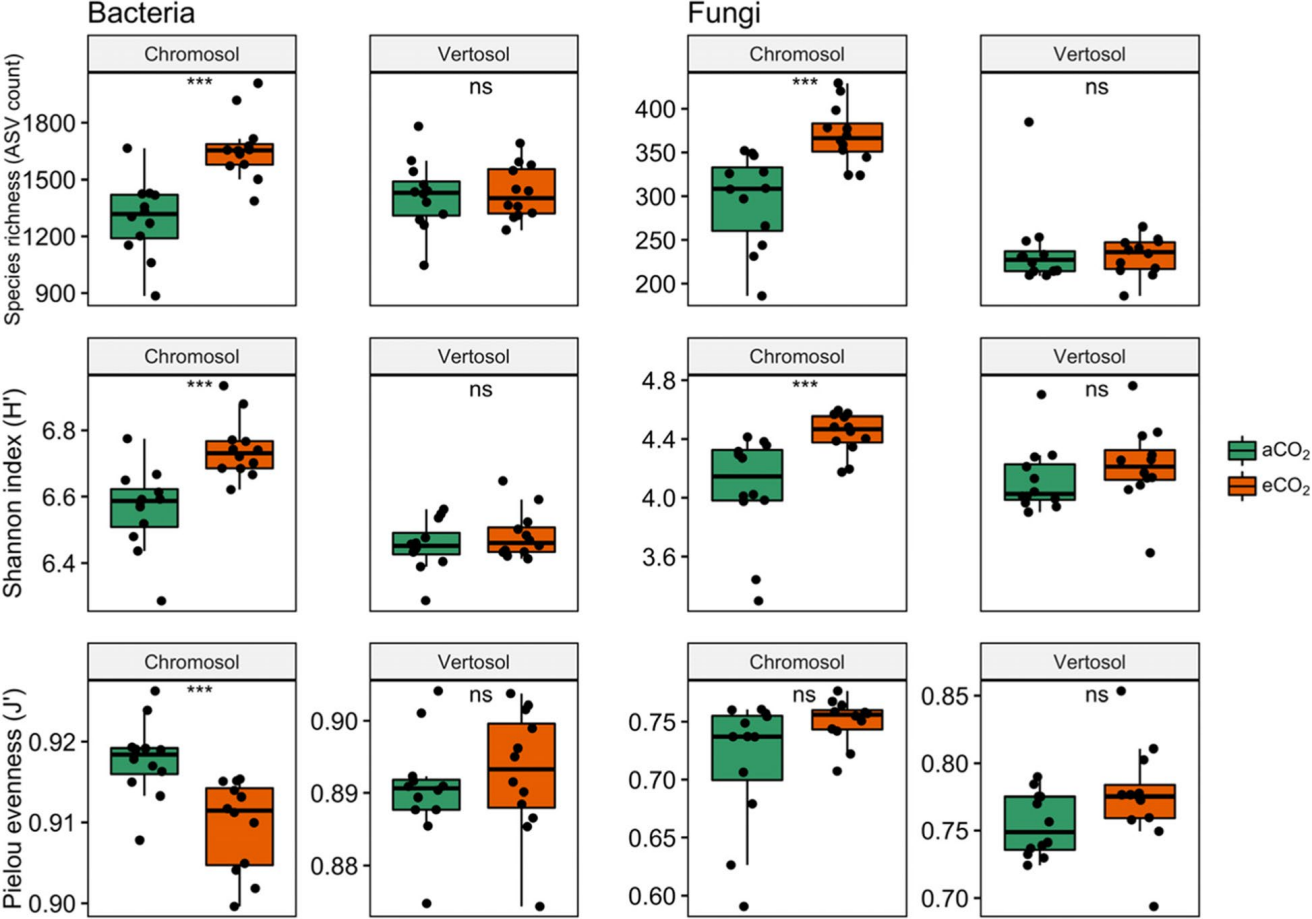
The observed bacterial and fungal species richness, Shannon and Pielou evenness indices across rhizosphere compartments of 0, 1.5, and 3 mm away from the root growth zone. Wheat plants were grown in Chromosol and Vertosol for 10 weeks under elevated CO_2_ (eCO_2_, 800 ppm) or ambient CO_2_ (aCO_2_, 400 ppm). The diversity indices were calculated from rarefied abundance of filtered amplicon sequence variants (ASVs). Bars show the maximum (top edge) and minimum (lower edge) percentiles, and boxes the 25% and 75% percentiles. The median (50%) percentile is represented by the horizontal line within the box. ns, *, **, and *** indicate significance of two-sample Wilcoxon Mann-Whitney tests at *p* > 0.05, *p* < 0.05, *p* < 0.01 and *p* < 0.001, respectively

Microbial diversity was more clearly affected by eCO_2_ in the Chromosol than in the Vertosol. Elevated CO_2_ significantly increased bacterial species richness by 33% across the 0, 1.5 and 3 mm rhizosphere compartments in the Chromosol (Fig. 4). A similar trend was observed with the Shannon diversity index which increased from 6.5 to 6.8 on average across the rhizosphere compartments in response to eCO_2_. The bacterial evenness in the rhizosphere of wheat grown in the Chromosol decreased under eCO_2_ relative to ambient CO_2_. In the Vertosol, however, species richness and Shannon and evenness indices in the rhizosphere were not significantly affected by eCO_2_.

Similar to the bacterial community, the fungal community had greater species richness and Shannon indices in the 0, 1.5, and 3-mm rhizosphere compartments when wheat plants were grown under eCO_2_ in the Chromosol (Figs. 4, S2). However, in the Vertosol, CO_2_ treatment did not significantly affect any indicators of α-diversity of fungal community.

**Fig. 5:**
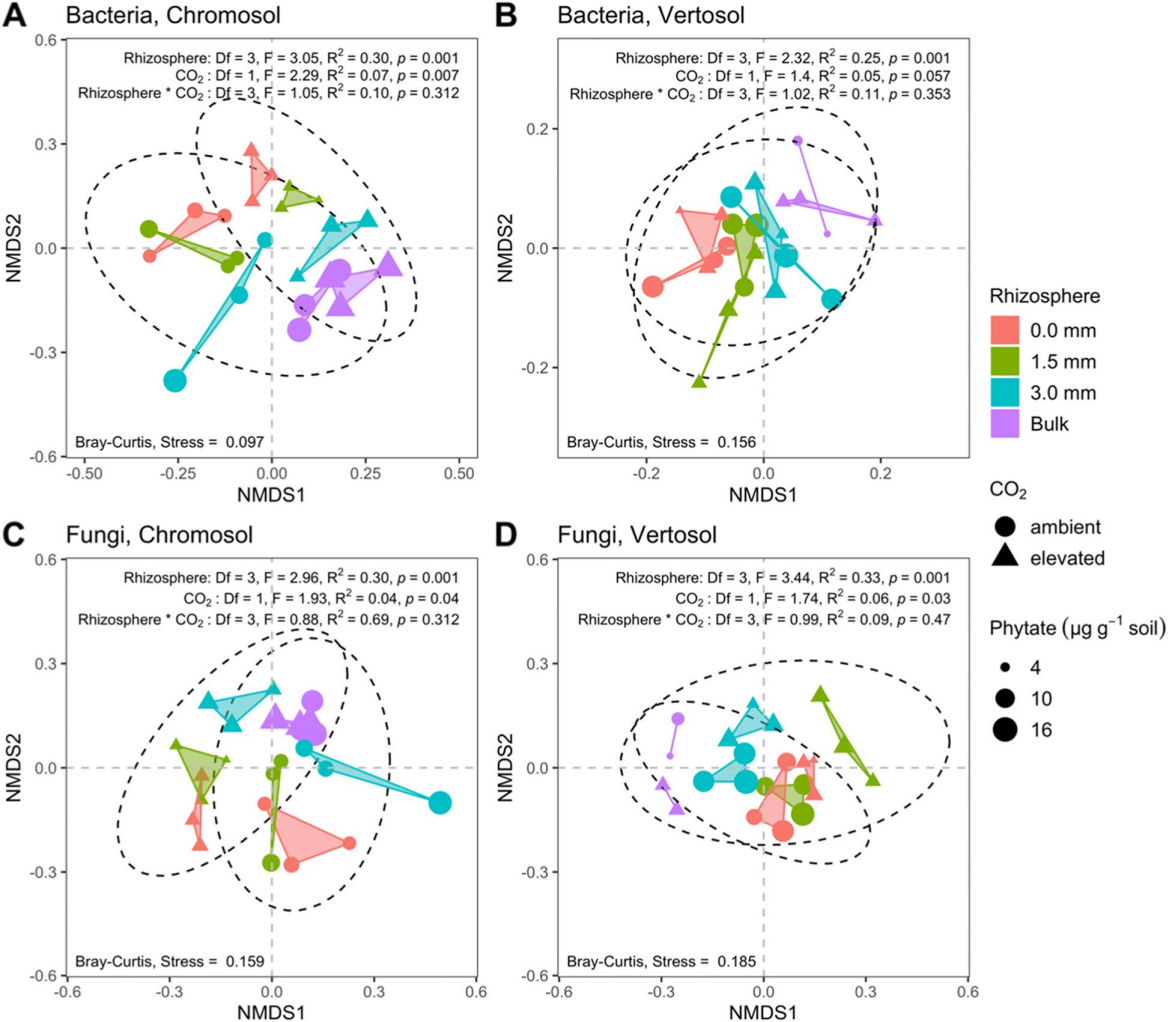
Non-metric multidimensional scaling (NMDS) of soil bacterial (**A** and **B**) and fungal (**C** and **D**) community composition (Bray-Curtis dissimilarities) in wheat rhizosphere compartments of 0, 1.5, and 3 mm away from the root growth zone and the bulk soil. Wheat plants were grown in Chromosol and Vertosol for 10 weeks under elevated CO_2_ (eCO_2_, 800 ppm) and ambient CO_2_ (aCO_2_, 400 ppm). Ellipses represent 95% confidence contours of samples grouped by ambient and elevated CO_2_. PERMANOVA results are presented based on Bray-Curtis dissimilarities for the main effects of rhizosphere and CO_2_ and their interaction on the soil community composition

The non-metric multidimensional scaling (NMDS) analysis further demonstrated that the bacterial and fungal communities in the rhizosphere zone differed from those in the bulk soil and had significant (*p* < 0.05) responses to eCO_2_ (Fig. 5) based on the test of permutational multivariate analysis of variance (PERMANOVA). The dissimilarities of the bacterial and fungal communities in the rhizosphere from 0 to 3 mm under eCO_2_ were clearly separated from those under aCO_2_ in the Chromosol, indicating that eCO_2_ changed microbial community composition (Fig. 5). The effect of eCO_2_ on microbial community composition in the Vertosol followed a similar trend.

**Fig. 6:**
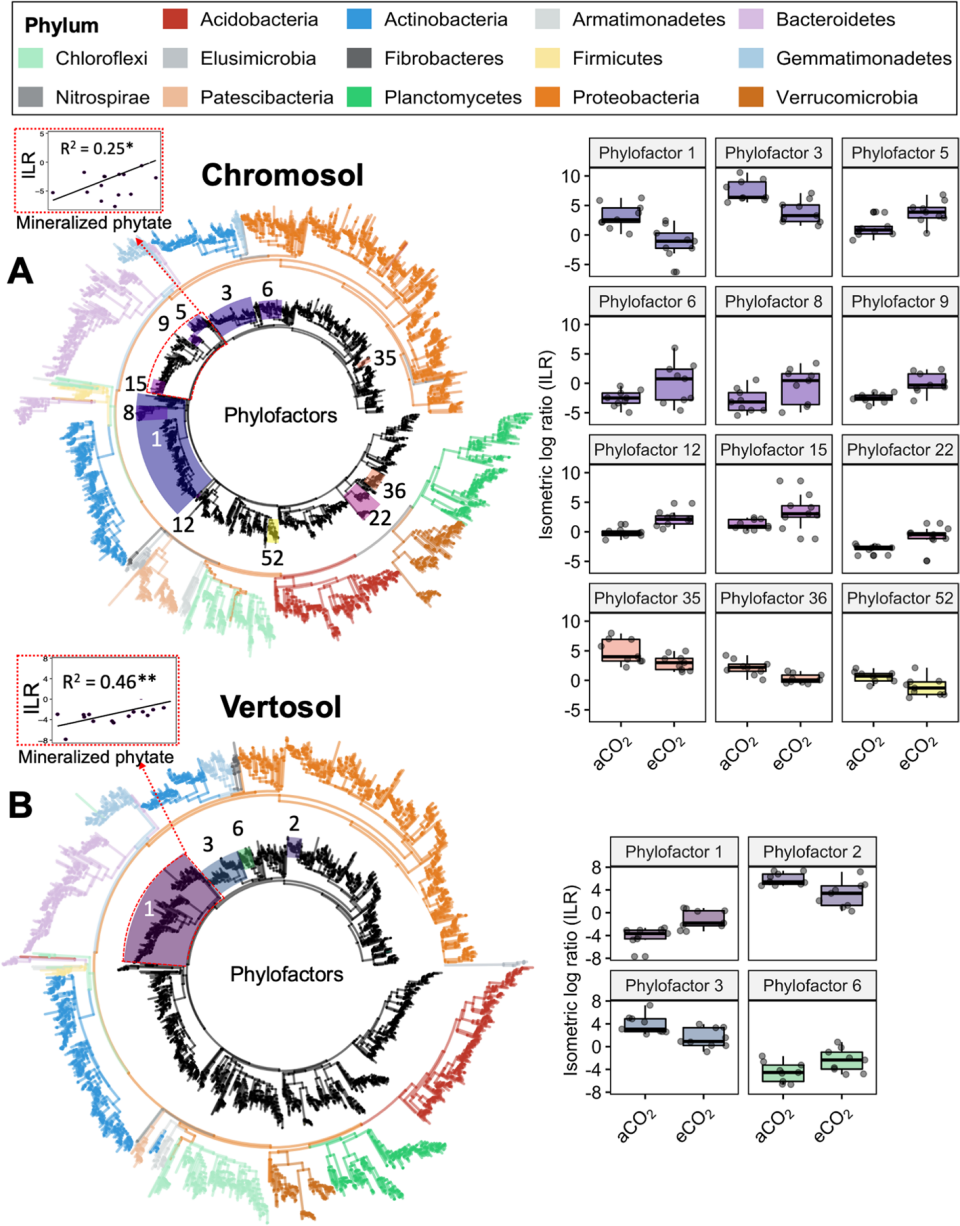
Effect of elevated CO_2_ on bacterial phylogenetic groups (phylofactors) based on modeling the isometric log ratios (ILR) of aggregated abundances as response and CO_2_ treatments (ambient or elevated) as explanatory variables (Model 1) using the package Phylofactor. Samples of all three rhizosphere compartments in Chromosol (**A**) and Vertosol (**B**) were combined (*n* = 18; Chromosol, *n* = 18; Vertosol). Edge colors in phylogenetic trees indicate their phylum affiliation (left tree). Highlighted groups (right tree) represent selected phylofactors with abundances that were associated with CO_2_. Boxplots (right) with ILRs of these phylofactors are presented. Taxa encased in dashed red rectangles highlight taxa which were associated with increased phytate mineralization according to a secondary phylofactor model (Model 2)

In order to present phylogenetic-scale-underlying patterns in the relative abundance data of ASVs in this study, we employed the phylofactor analysis to further confirm that both soils contained groups of Bacteroidetes which responded to eCO_2_ (Model 1) and were associated with increased phytate mineralization (Model 2) (Fig. 6). Through this analysis, the phylogenetic groups or phylofactors were identified and numbered based on the dominant axes of variation corresponding to contrasts of ASVs separated by an edge [25]. Phylofactor 8, 9 and 15 in the Chromosol, which represented the abundances of 112 ASVs in phylum Bacteroidetes, and phylofactor 1 in the Vertosol which represented the abundances of 270 ASVs mainly in phylum Bacteroidetes (and also included Gemmatimonadetes and Chloroflexi), all significantly responded to eCO_2_ (*p* < 0.05) (Fig. 6). The majority of these Bacteroidetes (96% of phylofactors 8, 9, and 15 of the Chromosol, 70% of phylofactor 1 of the Vertosol) belonged to families Sphingobacteriaceae, Chitinophagaceae, and Microscillaceae and in the case of the Vertosol also Hymenobacteraceae. In addition, phylofactor analysis showed that a group of Gemmatimonadaceae consisting of 47 ASVs in the Chromosol (Phylofactor 5) and a group of 105 ASVs in the Vertosol (Phylofactors 1 and 6) that mostly belonged to Gemmatimonadaceae, all significantly responded to eCO_2_ (*p* < 0.05). The relative abundances of Acidobacteria declined consecutively from the bulk soil to 3-, 1.5-, and 0-mm rhizosphere compartments while Bacteroidetes increased with proximity to roots (Fig. S3).

Moreover, in order to elucidate the association of microbial abundances with phytate mineralization, the regression-phylofactorization model [25] was adopted for the additive analysis of phylofactors which were regressed with mineralized phytate as an explanatory variable (Model 2). The results showed that the combined abundances of all Bacteroidetes and most Gemmatimonadetes were associated with increased phytate mineralization in the rhizosphere, a feature that was consistent across both soils (Phylofactor 1 in Chromosol and Vertosol) (Fig. 6).

**Fig. 7:**
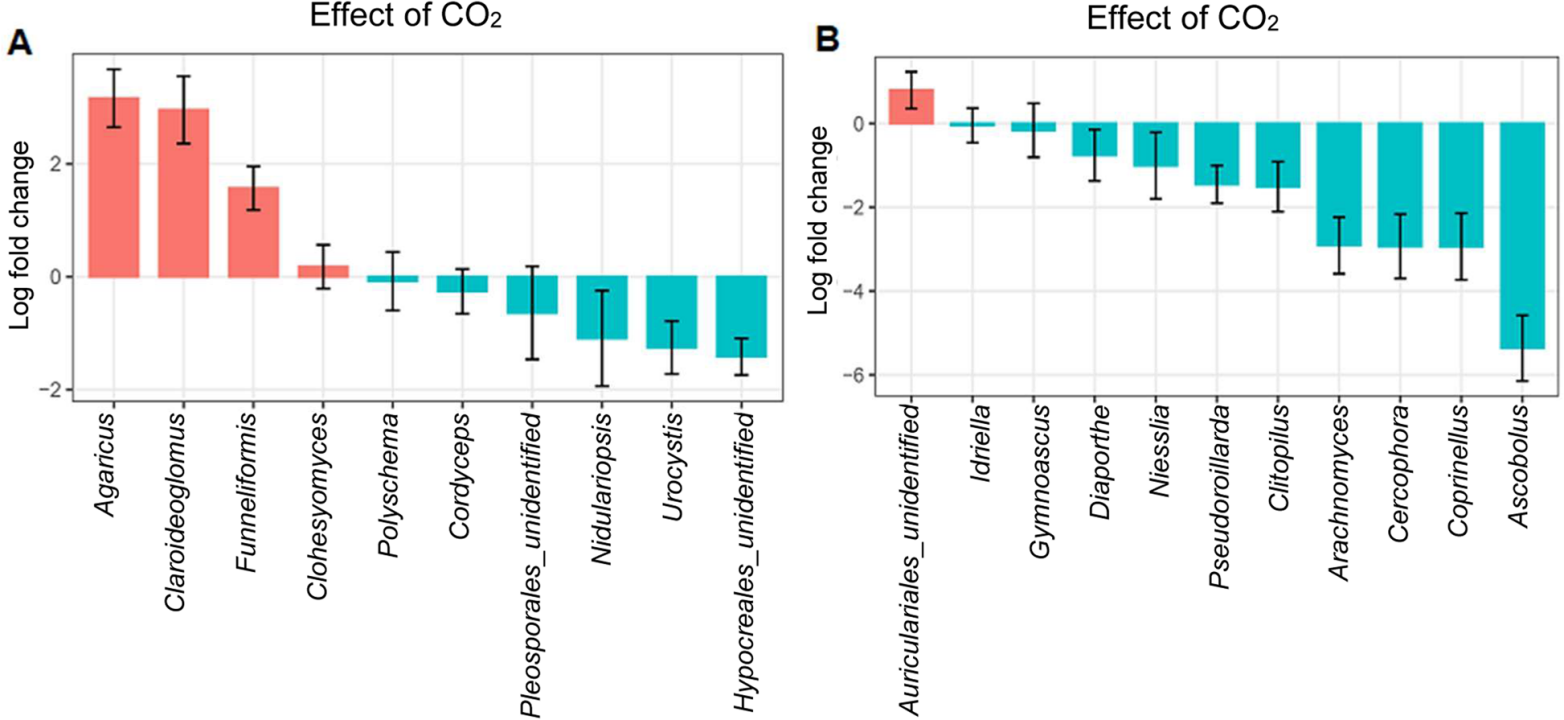
Effect of elevated CO_2_ on the abundance (centered-log ratios) of fungal genera in the rhizosphere. Wheat plants were grown in Chromosol (**A**) and Vertosol (**B**) for 10 weeks under elevated CO_2_ (800 ppm) and ambient CO_2_ (400 ppm). Genera responding significantly (*p* < 0.05, Holm corrected) to elevated CO_2_ with a log-fold change > 0.5 or < −0.5 are presented. Bars represent standard errors (*n*=9)

Across fungal genera, eCO_2_ significantly increased the abundances of Basidiomycota genus *Agaricus*, and the genera *Claroideoglomus* and *Funneliformis* of Glomeromycota in the rhizosphere of wheat in the Chromosol, and unidentified Basidiomycota fungi in the order *Auriculariales* in the Vertosol (Fig. 7).

### Microbial metabolic pathway in response to eCO_2_

**Fig. 8:**
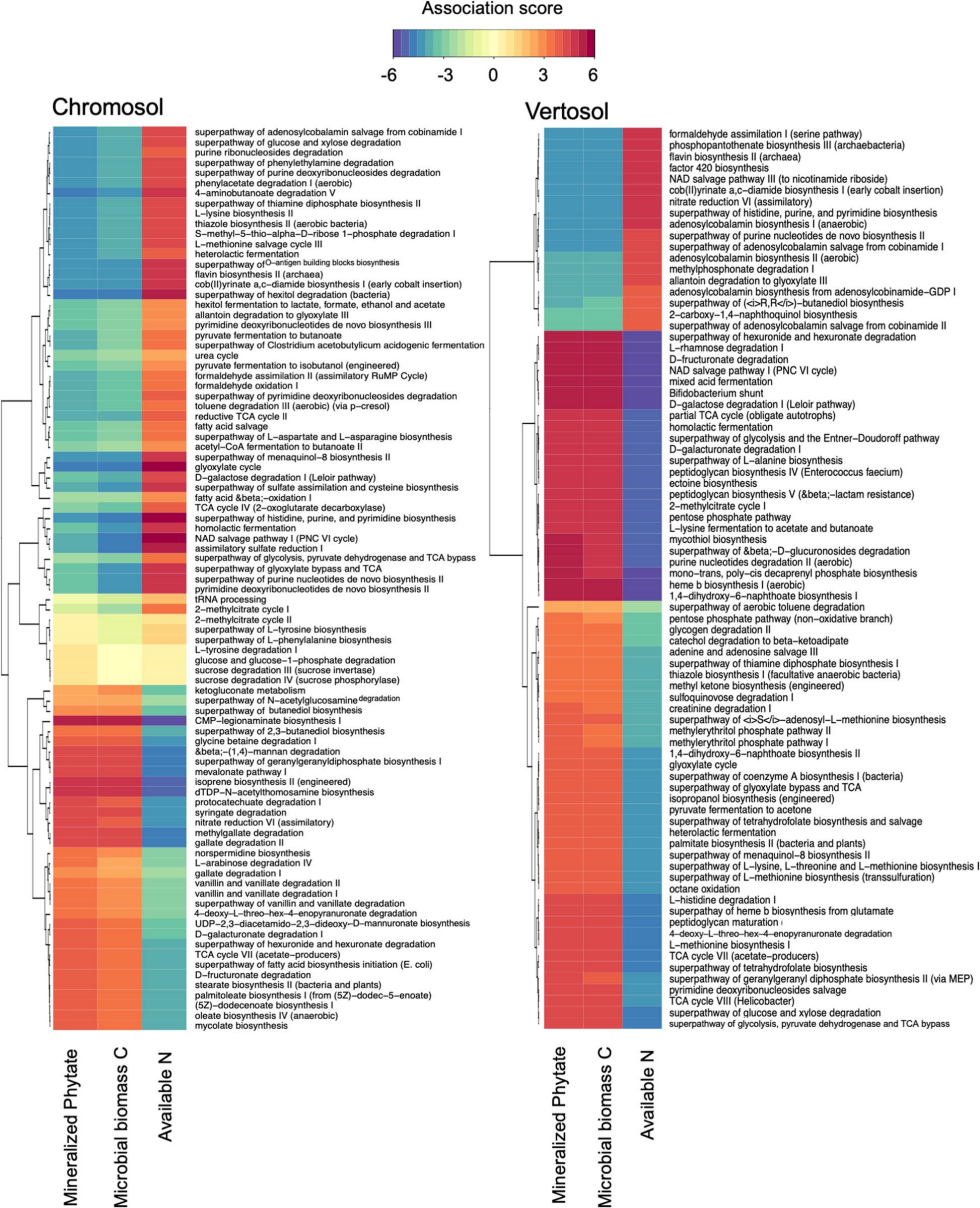
Heatmaps of relative abundances of microbial metabolic pathways (MetaCyc database) with soil variables of mineralized phytate, microbial biomass C, and available N across the rhizosphere compartments of wheat grown in Chromosol and Vertosol for 10 weeks under elevated CO_2_ (eCO_2_, 800 ppm) in comparison to ambient CO_2_ (aCO_2_, 400 ppm). The color indicates the strength of negative (blue to purple) or positive (orange to red) correlations of explanatory variables (pathway potentials) and response variables (soil variables) for the first two components of partial least squares regression. Dendrograms indicate distances of hierarchical clustering

It was estimated from bacterial marker genes that eCO_2_ significantly increased the genetic pool of bacteria for glycolysis (from glucose 6-phosphate) and for the pentose phosphate pathway in the Chromosol and Vertosol (Fig. S4). The pentose phosphate pathway generates pentoses and ribose 5-phosphate, a precursor for the synthesis of nucleotides, and the glycolysis pathway converts glucose to pyruvate, which produces high-energy adenosine triphosphate (ATP) [26, 27]. In addition, the metabolic potentials for salvage pathways of deoxyribonucleosides and ribonucleosides increased with eCO_2_ in the Chromosol and Vertosol (Fig. S4). As sparse partial least squares regression (sPLS) is an efficient method to deal with genomic selection data in terms of dimension reduction and variable selection [28], this approach has shown that in the Vertosol, the increases in the genetic potentials of glycolysis and the pentose phosphate pathways (among other metabolic pathways) were predictive of increased phytate mineralization (Fig. 8). Moreover, it was observed that in general, potentials of metabolic pathways that were predictive of increased phytate mineralization were associated with decreased available N and increased microbial C (Fig. 8).

## Discussion

### Increased microbial P demand favors P mineralization

Elevated CO_2_ raised the microbial P-use efficiency for population growth in the rhizosphere of wheat. The increased microbial P-use efficiency was attributable to the increase in bioavailable C in response to increased root exudation under eCO_2_. A greater amount of plant-derived C in the rhizosphere under eCO_2_ corresponded with increased microbial growth (Figs. 2 and 4), which is consistent with previous studies using other plant species [29–31]. Thus, the increase in microbial biomass within the rhizosphere resulted in more P demands (Fig. 2). However, the increased P-uptake by wheat plants due to eCO_2_ in this study (Fig. S1) likely enhanced the competition for P between plants and microbes in the rhizosphere, especially in P-deficient soils. The dramatic decrease in Olsen P concentration in the rhizosphere of wheat grown under eCO_2_ further indicated that microbial competitiveness for P in the rhizosphere would be enhanced (Fig. 1B). Consequently, the microbial C/P ratio in the rhizosphere considerably increased under eCO_2_ (Fig. 2), implying that the nutrient status of microorganisms in the rhizosphere turned from the C-limiting to P-limiting, and that the P-use efficiency of their population improved. Moreover, the increased respiration per unit microbial P under eCO_2_ further suggest that eCO_2_ improved the microbial P use at the metabolic level and this was most pronounced in the Vertosol.

The increase in microbial P-use efficiency under eCO_2_ may favor the mineralization of phytate. In this study, the mineralization of phytate was accelerated in the rhizosphere of wheat grown under eCO_2_ (Fig. 1), which is supported by previous FACE (Free Air CO_2_ Enrichment) studies reporting increased microbial access to the organic P fraction in a number of farming soils in response to eCO_2_ [10, 11]. Additionally, our present study showed that the increased phytate mineralization and the ensuing P-limitations to the microbial biomass (Figs. 2 and 3) favored the microbial population with a more efficient P metabolism in the rhizosphere. This was most obvious in the Vertosol where the response of phytate mineralization to the microbial C/P ratio was more significant (Fig. 3B).

### Bacterial taxonomic and metabolic attributes to P mineralization and microbial P-use efficiency

The actual mechanism of the eCO_2_-induced increase in phytate mineralization is about how the microbial community composition and function shifted in response to the increased plant C efflux into the rhizosphere under eCO_2_ and the amount of phytate in soil. In our study, β-diversity rather than α-diversity (species and abundance distributions on sample-level) of the microbial community corresponded with changes in phytate mineralization under eCO_2_. Species richness, Shannon’s diversity and Pielou’s evenness of bacteria did not change under eCO_2_ in the rhizosphere of wheat grown in the Vertosol, which was the more P-limiting soil (Fig. 4).

The β-diversity, on the other hand, was more responsive to P mineralization in the rhizosphere under eCO_2_. The results showed that eCO_2_ shifted the bacterial community composition in the rhizosphere of wheat which was significant in the Chromosol and marginally significant in the Vertosol (Fig. 5). Hence, it was suspected that across both soils some bacterial taxonomic groups with common metabolic traits responded to eCO_2_ and the ensuing P-limitations in the rhizosphere. Phylofactor analysis subsequently showed that in both the Chromosol and Vertosol, abundances of two major bacterial phyla, Bacteroidetes and Gemmatimonadetes were relatively enriched in the rhizosphere under eCO_2_ and positively associated with the mineralization of phytate (Figs. 6, S3). Taken together, this group of bacteria consisted of 375 ASVs (i.e., putative species) in the Vertosol and 159 ASVs in the Chromosol. Although those ASVs were different between the two soils, they likely performed similar metabolic functions regarding the C and P transformation, which will be discussed further below.

In this study, Bacteroidetes and Gemmatimonadetes were likely involved in the processes of C and P transformation in the rhizosphere under eCO_2_. The most prevalent Bacteroidetes families, Chitinophagaceae and Microscillaceae, followed by Sphingobacteriaceae and Hymenobacteraceae in this group may play such roles across both soils. For example, Chitinophagaceae have been found to be able to mineralize complex organic compounds such as chitin and cellulose [32, 33] and produce β-glucosidase [34]. Moreover, members of the Gemmatimonadaceae bacteria are adapted to low-nutrient environments and are known for their intracellular accumulation of polyphosphate and are indeed utilized for phosphate immobilization in wastewater treatment systems [33]. The similar response of Gemmatimonadaceae to organic P in the water and soil in this present study implies that Gemmatimonadacea play an important role for efficient phytate mineralization of the microbial population across various ecosystems.

A number of microbial families of the Bacteroidetes and Gemmatimonadetes associated with P mineralization had a similar response to eCO_2_ in the rhizosphere, indicating that similar phylotypes and genes may be involved in the eCO_2_ response. There were significant associations of P-mineralization with a number of functional genes coding for methylgallate, protocatchuate, and gallate degradation pathways in both soils (Fig. 8). These pathways are involved in the degradation of aromatic compounds as part of the terrestrial C cycle. As phytate is structured with the similar C skeleton to aromatic compounds, the mineralization of C and P might be a co-metabolic process. Spohn and Kuzyakov [35] also demonstrated that P mineralization in several forest soils was driven by microbial need for C, supporting the view of this point.

Elevated CO_2_ further altered the microbial metabolisms leading to an increase in microbial P-use efficiency. The metabolic mechanisms by which bacteria maximized their growth under eCO_2_, involved the central pathways for biosynthesis of RNA/DNA precursors. Phosphorus is a crucial constituent in nucleotides assembling to form nucleic acids. For both soils in this study, it was predicted from the ASV abundances that the genetic pool shifted towards the pentose phosphate pathway and pyrimidine deoxyribonucleoside salvage pathways under eCO_2_ (Fig. S4). The pentose phosphate pathway produces equivalents for anabolic reactions (Nicotinamide adenine dinucleotide phosphate (NDPH) and protects microbial cells against toxic reactive oxygen species (ROS). Furthermore, the de novo synthesis of ribonucleotides is an energy-consuming process and hence bacteria with an advantageous cell membrane morphology and the ability to express key enzymes to facilitate the pyrimidine deoxyribonucleoside salvage pathway under eCO_2_ have a distinct advantage. The results therefore indicated that when energy supply was abundant (e.g., increased root exudation under eCO_2_), such phylotypes became more competitive in the P-limiting environment through an increased ability to synthesize and maintain (phosphate-containing) DNA and RNA building blocks. The eCO_2_-induced shift of microbial metabolisms towards anabolic processes in the rhizosphere is likely to be an adaptative ability of microbial community to energy-enriched but P-deficient environments, which may result in the increase of microbial P-use efficiency under eCO_2_.

### Fungal community contribution to P mineralization

Greater fungal Shannon index was associated with increases in phytate mineralization in both the Chromosol and the Vertosol (Fig. S5). This implied a special role of fungi in the organic P mineralization under eCO_2_ in both soils. Under eCO_2_, the relative higher abundance of *Agaricus* in the Chromosol and *Auriculariales* in the Vertosol (Fig. 7), which are affiliated to Ectomycorrhiza, likely accessed diverse P pools and organic matters [36, 37]. Moreover, eCO_2_-induced enrichment of Claroideoglomus and Funneliformis in the rhizosphere in the Chromosol are affiliated with arbuscular mycorrhizas, which may improve the plant capability to compete for P in soil or stimulated bacteria-mediated organic P mineralization processes with increasing the expression of phosphatase genes in bacteria [38]. However, it is unclear whether the fungal community directly contributed to the phytate mineralization or accelerated the depletion of orthophosphate by plants via mycorrhizal hypha, resulting in the bacterial community change towards mining more organic P. In this study, the increase in the relative abundance of those fungal genera, i.e., root-colonizing arbuscular mycorrhizas, under eCO_2_ was positively associated with mineralized phytate and negatively with Olsen P, suggesting that the contribution of fungal community to the phytate mineralization might be indirectly via colonizing plant roots to deplete orthophosphate, and enhancing rhizobacteria to mineralize organic P.

Moreover, there were several more fungal genera associated with phytate mineralization which might be involved in the decomposition of organic matters for nutrients. In this study, *Ascobolus* and *Arachnomyces* were likely relevant to the phytate mineralization (Fig. 7), of which *Ascobolus* can access nutrients in dung [39] and *Arachnomyces* have been reported to be able to decompose lignin and cellulose [40]. Therefore, a number of genera in the fungal community might also directly accelerate phytate mineralization by decomposing organic compounds in the soil in the elevated CO_2_ environment.

## Conclusions

**Fig. 9:**
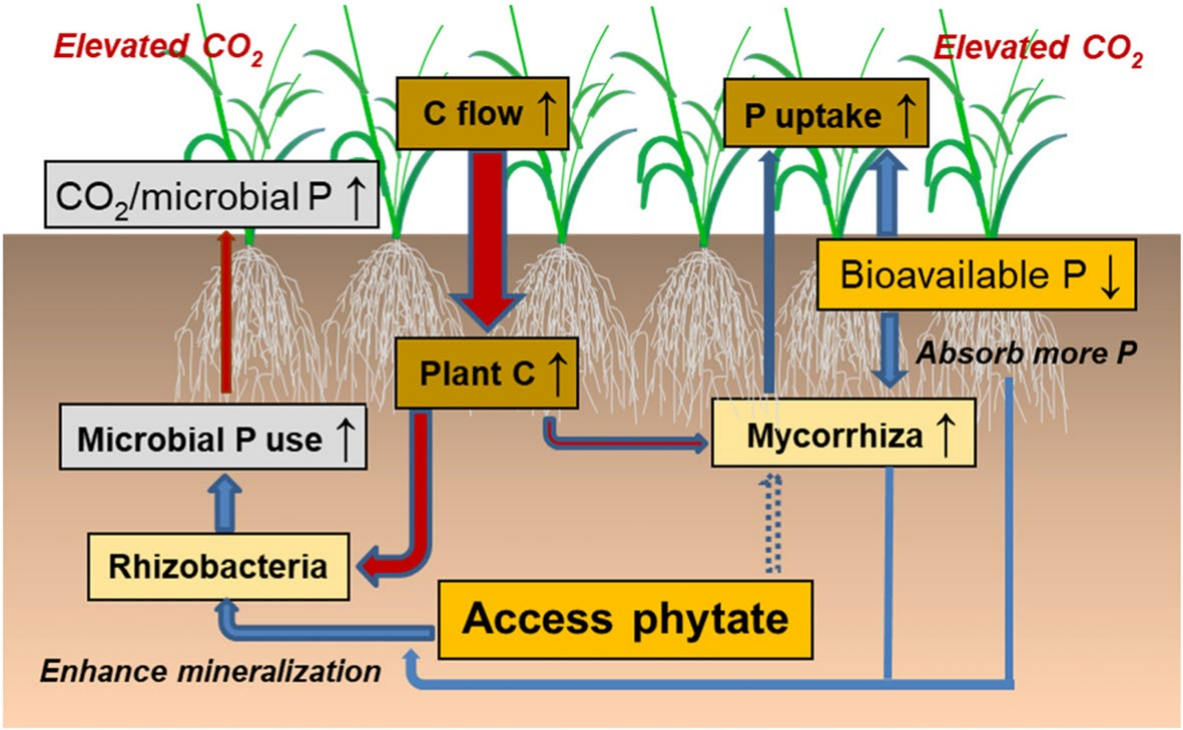
Diagram of microbial ability to access phytate in the rhizosphere of wheat grown under elevated CO_2_. The arrows (↑) and (↓) indicate increase and decrease, respectively. The red and blue arrows represent C flow and P dynamics, respectively. The dot arrow indicates unknown process of P transformation

Elevated CO_2_ accelerated the mineralization of phytate in the rhizosphere of wheat. Under eCO_2_ environments, the increased plant C efflux into the rhizosphere resulted in the stimulation of microbial activity, leading to stronger competition for P in the microbial community. The eCO_2_-induced increase in the relative abundances of the bacterial genera being able to degrade aromatic compounds likely contributed to the stimulation of phytate mineralization in the rhizosphere. Moreover, the enriched mycorrhizas might form a symbiotic relationship with plants to further deplete labile P in the rhizosphere, which enhanced the rhizobacterial capability to mineralize organic P (Fig. 9). The enhanced microbial anabolic metabolism under eCO_2_ may reflect an adaptative ability of the microbial community in the rhizosphere to respond to increasing plant-C flow under P-deficient conditions, resulting in the increases of microbial P-use efficiency and organic P mineralization. This microbial ability to metabolically mineralize organic P appears to be similar across the soils. The mineralization of organic P in soil is likely to be accelerated in the rhizosphere of crops grown under eCO_2_ environments due to the stimulation of microbial metabolisms on C and P transformation. Future study should clarify the link of bacterial and fungal integrative metabolisms with organic P mineralization in the rhizosphere under eCO_2_.

## Materials and methods

### *Experimental design*, *soils*, *and growth conditions*

A rhizobox experiment was established in growth cabinets where wheat (*Triticum aestivum*. L., cultivar Yitpi) was grown in two soils, Chromosol and Vertosol [41], under two CO_2_ concentrations (ambient CO_2_ (aCO_2_) and elevated CO_2_ (eCO_2_)) in a randomized block design. Twelve rhizoboxes were set up for each soil and planted with wheat, with six replicates grown under aCO_2_ and six under eCO_2_. Additionally, four no-plant controls were set up for each soil. Plant materials and rhizosphere soils were destructively harvested and processed after 10 weeks of growth.

The Chromosol soil was collected from the 0−15 cm topsoil layer of a long-term experimental site at the Agriculture Victoria research center in Hamilton, Victoria. (37° 50′ S, 142° 05′ E) [42]. The pasture plots, from which the soil was taken, had received an average of 15 kg P ha^-1^ year^-1^ in the form of triple superphosphate since 1977. Prior to the experiment, the soil had a total C of 43 g kg^-1^, total P of 200 mg kg^-1^, and Olsen P of 24.5 mg kg^-1^. The Vertosol soil was collected from the 0−15 cm topsoil at a long-term experiment run by the International Plant Nutrition Institute (IPNI) at Dahlen Victoria, Australia (36° 38′ S and 142° 08′ E). The long-term experiment was established in 1996. The plots were planted in a canola-wheat-barley-pulse rotation, received 80 kg N ha^1^ year^-1^ applied as urea and 18 kg P ha^-1^ year^-1^ applied as triple superphosphate. The Vertosol had a total soil C of 12.2 g kg^-1^, total P 172 mg kg^-1^, and Olsen P 15.0 mg kg^-1^. Other basic soil physicochemical properties are shown in Table S1.

Uniform wheat seeds were selected for 1-day germination on moistened filter paper under 25°C. Each rhizobox was sown with six geminated seeds and five plants were kept after emergence. All rhizoboxes were allocated into four plant growth cabinets (Fitotron SGC120, Weiss Gallenkamp Ltd., Leicestershire, UK) with two cabinets set at aCO_2_ (400 ppm) and two at eCO_2_ (800 ppm). The eCO_2_ concentration selected was in the range of predictive levels by the end of this century (IPCC 2021). Temperature was set at 22°C (day, 12 h) and 18°C (night), and light intensity (over the waveband 400–700 nm) was at 550 μmol photons m^−2^ s^−1^. Soil water content was maintained at 80±5% of field capacity. Two watering tubes were installed in the bulk soil on each side of rhizospheric compartments in each rhizobox. Via these tubes, the deionized water was added into soil daily to achieve the designed soil moisture uniformly in soil by weighing.

### Rhizobox setup

The rhizobox was constructed for collecting rhizosphere soil at precise distances from the root growth zone, which was developed by Youssef and Chino [43]. The dimension of the rhizobox was 120 mm in width, and 200 mm in length and height. Each rhizobox comprised two compartment types, i.e., root growth and rhizosperic compartments, which vertically sat inside of the rhizobox. Two rhizospheric compartments were attached to each side of the root-growth compartment (Fig. S6). The thicknesses of root-growth and rhizospheric compartments were 3 mm and 1.5 mm, respectively. Each compartment was separated by PVC frames covered with nylon cloth (mesh size < 26 μm), which allows the movement of water, nutrients, and microorganisms across the compartments.

Phytate (C_6_H_6_O_24_P_6_Ca_6_, Sigma Aldrich, USA) at a rate of 70 mg P kg^-1^ soil was mixed with soil thoroughly before loaded into compartments. Basal nutrients were simultaneously added as well in the following composition (mg kg^-1^): urea, 60; K_2_SO_4_, 147; CaCl_2_, 186; MgSO_4_.7H_2_O, 122; MnSO_4_.H_2_O, 6; ZnSO_4_.7H_2_O, 8; CuSO_4_.5H_2_O, 6; CoCl_2_.6H_2_O, 0.4; FeCl_3_, 0.6; NaB_4_0_7_.10H_2_O, 1.6 and Na_2_MoO_4_.2H_2_O, 0.4.

### ^13^CO_2_ labeling

Wheat plants were labeled with ^13^CO_2_ for 10 days in air-tight clear plastic chambers (area 500 × 540 mm^2^, height 680 mm) prior to harvest. ^13^CO_2_ concentrations of 500 and 900 ppm were attained for aCO_2_ and eCO_2_, respectively, after injection of 9.2 M sulfuric acid (H_2_SO_4_) into Na_2_^13^CO_3_ inside the chamber (Fig. S6). Once the CO_2_ concentration dropped by 200 ppm, new injections occurred to maintain the ^13^CO_2_ levels to an average of 400 and 800 ppm over a 6-h labeling period per day. The frequency of injection depended on the reduction rate of ^12^CO_2_ inside the chambers in a preliminary test using a CO_2_ meter [44]. A fan was placed inside each chamber to homogenize the air. During labeling, half of the pots in each treatment were sealed in chambers while the other half were not subjected to ^13^CO_2_ labeling as unlabeled controls.

### Harvest and biochemical measurements

After 10 weeks of plant growth, wheat plants were harvested by cutting shoots at the ground level and processed according to Tang et al*.* [45]. The rhizosphere compartments were carefully pulled out of the rhizobox and dismantled into five individual compartments: one root-growth compartment at 0 mm, and two rhizospheric compartments at 1.5 mm and the other two at 3 mm away from both sides of the root-growth compartment (Fig. S6). Soil samples were collected from the rhizospheric compartments and considered as rhizosphere soil. Soils in those compartments at the same distance from the root-growth compartment on both sides were combined and homogenized. Approximately 5 g of soil was immediately put into liquid nitrogen for 15 min, and then stored at −80°C for DNA extraction. A sample of soil (40 g) was kept at 4°C for available N, microbial biomass and respiration measurements while approximately 30 g of soil was air-dried for measurements of chemical properties.

Shoots and roots were washed and dried at 70°C for 72 h and dry weights recorded. Plant materials were finely ground using a ball mill (Retsch MM400, Germany), and C and P concentrations were measured using a dry-combustion analyzer (PerkinElmer EA2400, Shelton, CT, USA), and a spectrometer [46] after acid digestion [47].

Air-dried soil samples were used to measure Olsen P concentration according to Olsen et al. [48], and pH using a Wettler Toledo 320 pH meter (soil to water = 1:5). The stable ^13^C isotope ratios in soil and plant samples were analyzed with an isotope ratio mass spectrometer (Delta^plus^, Finnigan MAT GmbH, Bremen, Germany). To measure phytate concentrations, 1 g soil was extracted with 0.1 mol L^-1^ NaOAc with 50 mmol L^-1^ ethylenediaminetetraacetic acid (EDTA) for 4 h of shaking before centrifuged at 5000 *g* for 20 min. Afterwards 1 mL of extract was added to 1 mL of 0.1 mol L^-1^ sodium acetate together with wheat phytase (20 g L^-1^) (Sigma Aldrich) and incubated at 55°C overnight. An equal volume of 1 mol L^-1^ hydrochloric acid was used to cease reactions. Phytate P concentrations were quantified as inorganic P after hydrolysis minus the controls without addition of phytase [49, 50]. The malachite green method was used to measure the concentration of inorganic P in solutions [51].

With fresh soil samples, NH_4_^+^ and NO_3_^-^ were determined by a continuous flow analytical system (SKALAR SAN^++^, Skalar, the Netherlands) after extraction in 2 M KCl for 1 h [52]. The microbial biomass carbon (MBC) was calculated as the difference in total organic C (TOC) concentrations in extracts between fumigated and non-fumigated soils [53]. The TOC concentration in the non-fumigated control was considered as dissolved organic C (DOC) [54]. Microbial P was measured according to Brookes et al*.* [55]. Using an Infra-red Gas Analyzer (Servomex 4210 Industrial Gas Analyzer, Cowborough, UK) [56, 57], microbial respiration was measured after 10 g fresh soil was incubated in a sealed half-pint (237 ml) Mason jar at 25°C for 24 h.

### Marker gene sequencing and bioinformatic processing

Soil DNA was extracted using a DNeasy Powersoil Pro extraction kit (Qiagen, Hilden, Germany) and stored at −20°C. DNA extracts were dissolved in 100 μl of TE buffer (10 mM Tris-HCl, 1 mM EDTA, pH 8.0). The V4 hypervariable region of bacterial 16S rRNA genes were amplified with the primer pair 515F (GTGYCAGCMGCCGCGGTAA)/806R (GGACTACNVGGGTWTCTAAT) [58]. The Internal Transcribed Spacer (ITS) region 2 of fungi was amplified with primers FITS7 (GTGARTCATCGAATCTTTG)/ITS4 (TCCTCCGCTTATTGATATGC) [59]. Library preparation was performed for 16S and ITS amplicon sequencing using Nextera XT indices according to Illumina reference guides and then sequenced on an Illumina MiSeq platform (2 × 300) [60, 61].

The default pipeline settings of Quantitative Insights Into Microbial Ecology (QIIME2) (version 2020.2; http://qiime.org/) including the plugins cutadapt [62] and dada2 [63] were deployed to assess read quality, trim primers, denoise, filter, and dereplicate sequences. The sequences were truncated to 290 bp. A total of 3,017, 587 and 942,797 reads were retained at a median frequency of 53,362 and 16,955 per sample for bacteria and fungi, respectively. Primer specific taxonomy classifiers were then used (Silva132 at 99% similarity and UNITE v8 dynamic) to assign taxonomies to amplicon sequence variants (ASVs) with the qiime2 plugin “classify- sklearn.” Amplicon sequence variants with less than 10 reads were filtered out resulting in a total of 5 565 bacterial and 1 562 fungal ASVs for diversity analyses.

Enzyme metagenomes and abundances of metabolic pathways (MetaCyc database) [64] were predicted from bacterial ASVs using PICRUSt2 (Phylogenetic Investigation of Communities by Reconstruction of Unobserved States 2) [65] with default settings. ASVs that were not present in at least two samples and with a frequency of less than 10 reads were removed. The filtered abundance table comprised of 423 pathways.

### Diversity and statistical analyses

Analysis of variance (ANOVA) with a block design model [66] in Genstat 19 (VSN International, Hemel Hemspstead, UK) was used to estimate the effects of eCO_2_ and rhizosphere, and their interactions on the concentrations of phytate, available N (NO_3_^-^ and NH_4_^+^), DOC, and Olsen P, MBC, microbial P, microbial C/P, microbial respiration, and microbial respiration per unit microbial P.

The α-diversity indices were calculated after rarefying abundances to a depth of minimum sample size (27,038 and 6262 reads for bacteria and fungi, respectively). Common diversity indices such as species richness (N0) and Shannon index (H') were calculated with function “estimate_richness” from the Phyloseq package [67] while Pielou evenness (J') was calculated as following [68]:

$${J}^{\prime }=\frac{H^{\prime }}{\ln (N0)}$$

Differences in β-diversity of bacterial and fungal community compositions were visualized on two axes with nonmetric dimensional scaling (NMDS) with Bray-Curtis dissimilarities using the “ordinate” function of the Phyloseq package [67]. Permutational multivariate analysis of variance (PERMANOVA) was then performed using the “adonis” function of the vegan package [69] on the same dissimilarities to assess significant differences of community composition between the rhizosphere compartments and CO_2_ treatments.

Moreover, phylofactorization was performed to study associations of eCO_2_ and mineralized phytate with taxonomic groups of bacteria. Bacterial ASVs were regressed into phylogenetic factors based on significant F-statistics at the adjusted *p* < 0.05 level using ratios of relative abundances as response variable and either CO_2_ treatments (Model 1) or mineralized phytate (Model 2) as explanatory variables. First, a phylogenetic tree was created using the default options of the Qiime2 plugin q2-fragment-insertion with the silva reference tree (sepp-refs-silva-128, https://docs.qiime2.org/2020.11/data-resources). Relative abundances which were transformed to isometric log ratios (ILR) were then regressed along edges of the phylogenetic tree with the package phylofactor [25] as described in the package tutorial (https://github.com/reptalex/phylofactor) after pruning the data set to rhizosphere samples only (*n* = 18 per soil per soil type) and filtering amplicon sequence variants (ASVs) to keep those with a minimum of 50 reads (to minimize undue influence of minor abundances on phylofactors). Briefly, the package phylofactor was applied to break apart the phylogeny with a variety of contrasts and objective functions, summarize the splits, and visualize the circle tree with ASVs generated from rhizosphere soils. Selected phylogenetic groups which were associated with CO_2_ or mineralized phytate were visualized on a phylogenetic tree and the corresponding ILRs of their abundances (i.e., the ratio from contrast basis elements of abundances of two taxonomic groups) were plotted.

Phylogenetic methods for fungal ITS-based amplicons were not used in this study. Instead, analysis of Compositions of Microbiomes with Bias Correction (ANCOM-BC) [70] was deployed to evaluate the effect of CO_2_ treatment on the abundance of fungal genera using the package ANCOMBC.

Lastly, the mixOmics package was utilized for sparse partial least squares (sPLS) regression (http://mixomics.org/methods/spls) to predict mineralized phytate, microbial C, and available N (Y matrix) from Picrust2-predicted metabolic pathway abundances in the rhizosphere as explanatory variables (X matrix). Heatmaps of correlation clusters between important pathways and mineralized phytate, microbial C, and available N of the first two regressed components were subsequently created to visualize their associations. Prior to analysis, the pathway potentials were pruned to those pathways with a minimum relative abundance of 0.011% resulting in a total of 331 pathways. Furthermore, Wilcoxon Mann-Whitney tests were performed on relative abundances (centered log ratio transformed) of each pathway (*n* = 18/soil) to identify the effect of eCO_2_ on the bacterial metabolic potentials in the rhizosphere. Accordingly, metabolic pathways that increased in both soils as a result of eCO_2_ were selected for further review.

## Supplementary Information

**Additional file 1: Figure S1** The biomass of (A), and P contents (B), and ^13^C atom‰ excess in shoot and root (C), and soil ^13^C atom‰ excess in the rhizosphere compartments of 1.5 and 3 mm away from the root growth zone (D). Wheat plants were grown in Chromosol and Vertosol for 10 weeks under elevated CO_2_ (eCO_2_, 800 ppm) and ambient CO_2_ (aCO_2_, 400 ppm). Error bars are standard errors (*n*=6). *, ** and *** indicate significant differences between aCO_2_ and eCO_2_ treatments at *p* < 0.05, *p* < 0.01 and *p* < 0.001, respectively. The significance levels of the main effects of CO_2_ and rhizosphere (Rhizo) and their interactions on ^13^C atom‰ excess in Chromosol and Vertosol are presented. **Figure S2** Boxplots of species richness, Shannon indices and Pielou evenness in the rhizosphere compartments at 0 mm, 1.5 mm and 3 mm, and in the bulk soil of Chromosol and Vertosol. Wheat plants were grown in Chromosol and Vertosol for 10 weeks under elevated CO_2_ (eCO_2_, 800 ppm) and ambient CO_2_ (aCO_2_, 400 ppm). **Figure S3** The effects of elevated CO_2_ (A, C) and rhizosphere (B, D) on the relative abundances of bacterial phyla in Chromosol (A, B) and Vertosol (C, D). Wheat plants were grown for 10 weeks in a rhizobox comprising rhizosphere compartments of 0, 1.5 and 3 mm away from the root growth zone and the bulk soil under elevated CO_2_ (eCO_2_, 800 ppm) and ambient CO_2_ (aCO_2_, 400 ppm). **Figure S4** Relative abundances (centred log-ratios) of selected microbial metabolic pathways in the rhizosphere of wheat. Plants were grown in Chromosol and Vertosol under elevated CO_2_ (eCO_2_, 800 ppm) and ambient CO_2_ (aCO_2_, 400 ppm) for 10 weeks. ns, *, ** and *** indicate significance of two-sample Wilcoxon Mann-Whitney tests at *p* > 0.05, *p* < 0.05, *p* < 0.01 and *p* < 0.001, respectively. **Figure S5** Scatterplots of mineralized phytate (μg g^-1^) to species richness, Shannon indices and Pielou evenness in the rhizosphere compartments of 0 mm, 1.5 mm and 3 mm, and the bulk soil of Chromosol and Vertosol. **Figure S6** The photos showing (A) plants grown for 10 weeks under ambient CO_2_ (aCO_2_, 400 ppm) and elevated CO_2_ (eCO_2_, 800 ppm), and (B) a rhizo-compartment, and (C) a schematic structural diagram of the rhizobox in the ^13^C-labelling device used in the experiment. **Table S1** The concentrations of total organic C, total P and Olsen P, soil pH and soil particle composition (texture) of the Chromosol and Vertosol used in the study

## Data Availability

Please contact author for data requests.

## Abbreviations

C: Carbon
P: Phosphorus
DOC: Dissolved organic C
TOC: Total organic C
aCO_2_: Ambient CO_2_
eCO_2_: Elevated atmospheric CO_2_
FACE: Free air CO_2_ enrichment
ASVs: Amplicon sequence variants
NMDS: Non-metric multidimensional scaling
PERMANOVA: Permutational multivariate analysis of variance
ANOVA: Analysis of variance
ILR: Isometric log ratios
sPLS: Sparse partial least squares regression
ATP: Adenosine triphosphate
NDPH: Nicotinamide adenine dinucleotide phosphate
ROS: Reactive oxygen species
ITS: Internal transcribed spacer
ANCOM-BC: Compositions of microbiomes with bias correction
