# Supplementary material for: Elevated atmospheric CO2 alters the microbial community composition and metabolic potential to mineralize organic phosphorus in the rhizosphere of wheat
Source: Microbiome. 2022 Jan 24;10:12. doi: 10.1186/s40168-021-01203-w (PMC8785599; doi:10.1186/s40168-021-01203-w)
Supplement: Supplementary file 2 — Additional file 1: Figure S1 The biomass of (A), and P contents (B), and 13C atom‰ excess in shoot and root (C), and soil 13C atom‰ excess in the rhizosphere compartments of 1.5 and 3 mm away from the root growth zone (D). Wheat plants were grown in Chromosol and Vertosol for 10 weeks under elevated CO2 (eCO2, 800 ppm) and ambient CO2 (aCO2, 400 ppm). Error bars are standard errors (n=6). *, ** and *** indicate significant differences between aCO2 and eCO2 treatments at p < 0.05, p < 0.01 and p < 0.001, respectively. The significance levels of the main effects of CO2 and rhizosphere (Rhizo) and their interactions on 13C atom‰ excess in Chromosol and Vertosol are presented. Figure S2 Boxplots of species richness, Shannon indices and Pielou evenness in the rhizosphere compartments at 0 mm, 1.5 mm and 3 mm, and in the bulk soil of Chromosol and Vertosol. Wheat plants were grown in Chromosol and Vertosol for 10 weeks under elevated CO2 (eCO2, 800 ppm) and ambient CO2 (aCO2, 400 ppm). Figure S3 The effects of elevated CO2 (A, C) and rhizosphere (B, D) on the relative abundances of bacterial phyla in Chromosol (A, B) and Vertosol (C, D). Wheat plants were grown for 10 weeks in a rhizobox comprising rhizosphere compartments of 0, 1.5 and 3 mm away from the root growth zone and the bulk soil under elevated CO2 (eCO2, 800 ppm) and ambient CO2 (aCO2, 400 ppm). Figure S4 Relative abundances (centred log-ratios) of selected microbial metabolic pathways in the rhizosphere of wheat. Plants were grown in Chromosol and Vertosol under elevated CO2 (eCO2, 800 ppm) and ambient CO2 (aCO2, 400 ppm) for 10 weeks. ns, *, ** and *** indicate significance of two-sample Wilcoxon Mann-Whitney tests at p > 0.05, p < 0.05, p < 0.01 and p < 0.001, respectively. Figure S5 Scatterplots of mineralized phytate (μg g-1) to species richness, Shannon indices and Pielou evenness in the rhizosphere compartments of 0 mm, 1.5 mm and 3 mm, and the bulk soil of Chromosol and Vertosol. Figure S6 The photos sh [file 40168_2021_1203_MOESM2_ESM.docx]

# Supplementary materials

**Figure S1** The biomass of (A), and P contents (B) and ^13^C atom‰ excess in shoot and root (C), and soil ^13^C atom‰ excess in the rhizosphere compartments of 1.5 and 3 mm away from the root growth zone (D). Wheat plants were grown in Chromosol and Vertosol for 10 weeks under elevated CO_2_ (eCO_2_, 800 ppm) and ambient CO_2_ (aCO_2_, 400 ppm). Error bars are standard errors (n=6). *, ** and *** indicate significant differences between aCO_2_ and eCO_2_ treatments at *p* < 0.05, *p* < 0.01 and *p* < 0.001, respectively. The significance levels of the main effects of CO_2_ and rhizosphere (Rhizo) and their interactions on ^13^C atom‰ excess in Chromosol and Vertosol are presented.


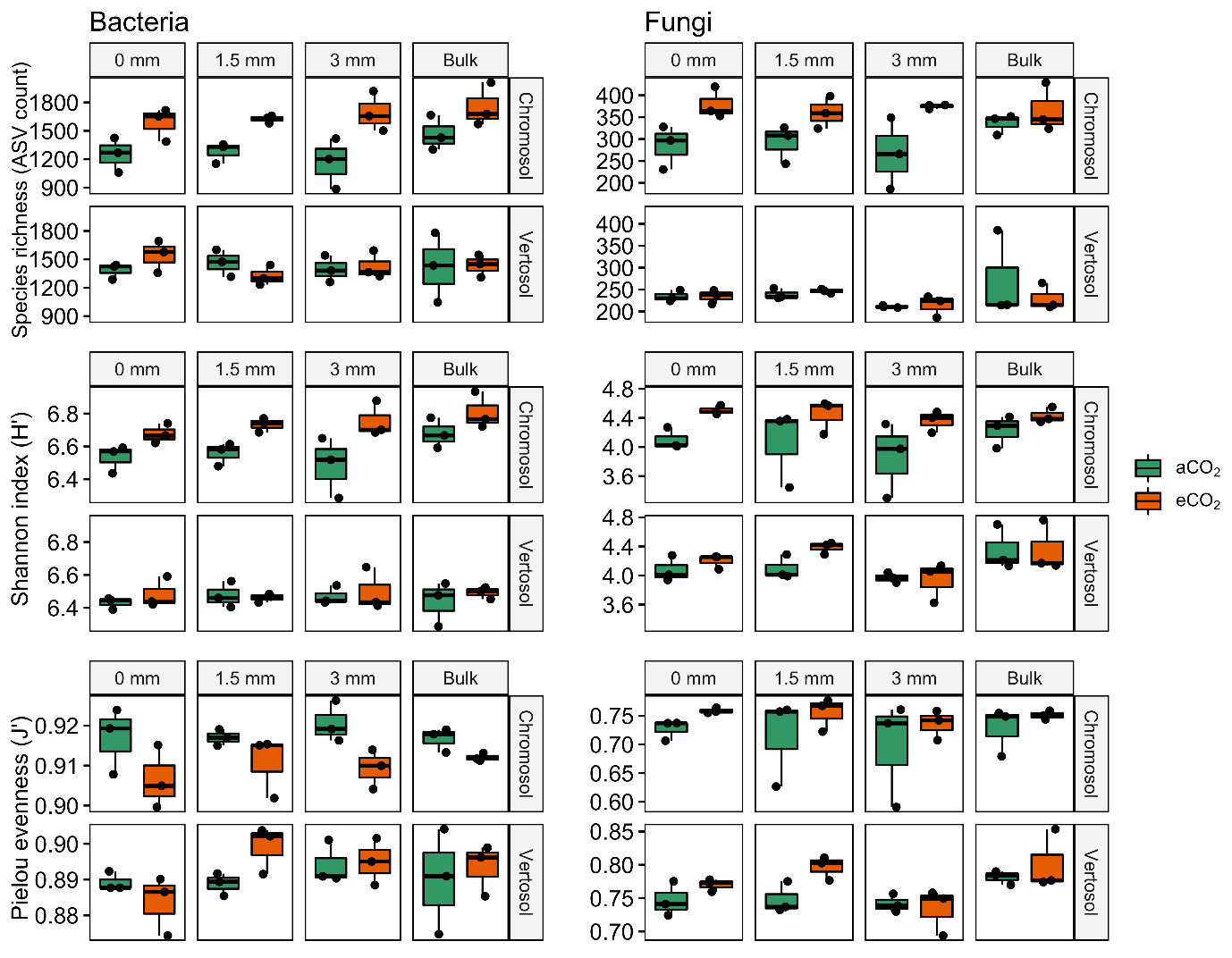


**Figure S2** Boxplots of species richness, Shannon indices and Pielou evenness in the rhizosphere compartments at 0 mm, 1.5 mm and 3 mm, and in the bulk soil of Chromosol and Vertosol. Wheat plants were grown in Chromosol and Vertosol for 10 weeks under elevated CO_2_ (eCO_2_, 800 ppm) and ambient CO_2_ (aCO_2_, 400 ppm).

**
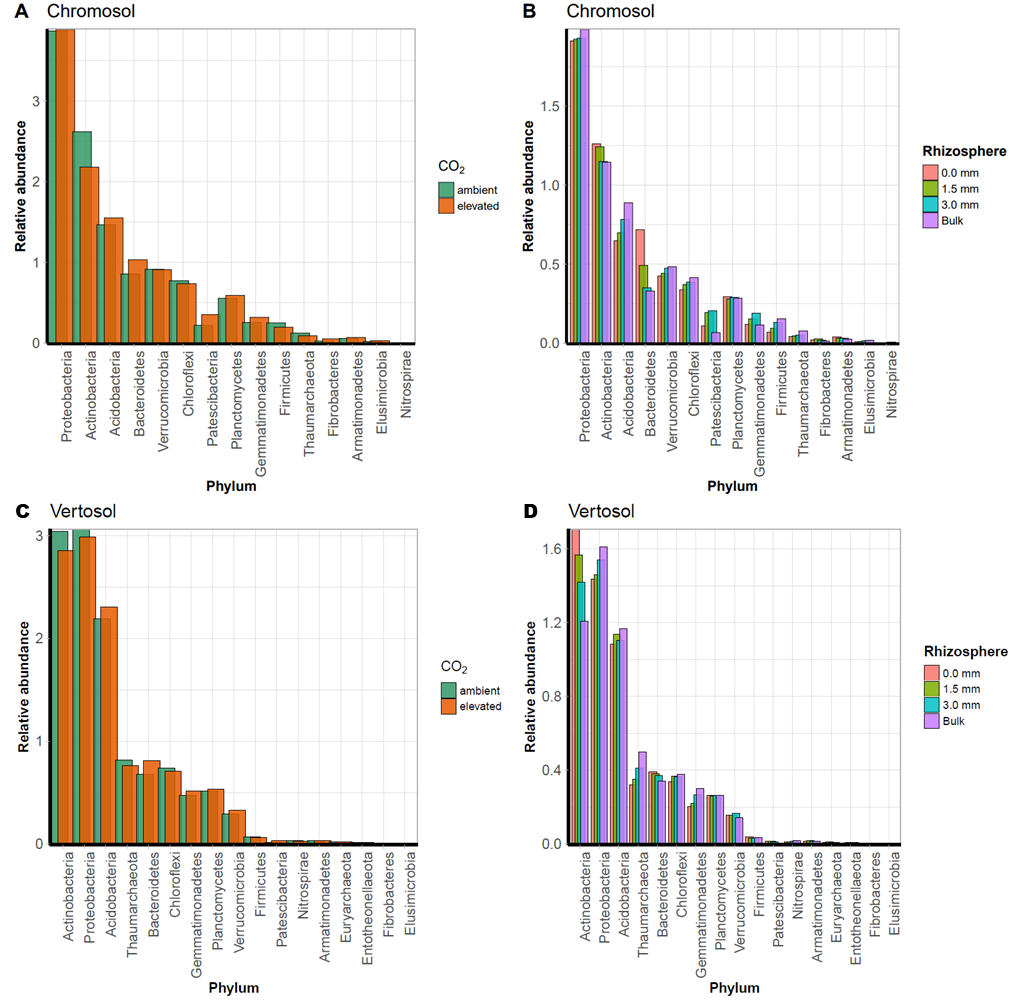
**

**Figure S3** The effects of elevated CO_2_ (A, C) and rhizosphere (B, D) on the relative abundances of bacterial phyla in Chromosol (A, B) and Vertosol (C, D). Wheat plants were grown for 10 weeks in a rhizobox comprising rhizosphere compartments of 0, 1.5 and 3 mm away from the root growth zone and the bulk soil under elevated CO_2_ (eCO_2_, 800 ppm) and ambient CO_2_ (aCO_2_, 400 ppm).


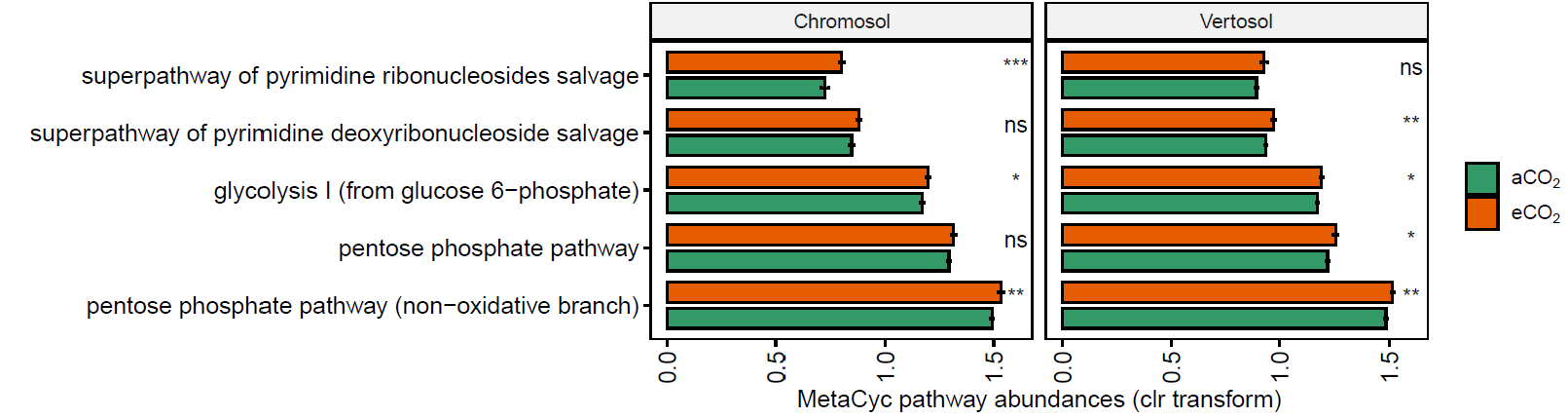


**Figure S4** Relative abundances (centred log-ratios) of selected microbial metabolic pathways in the rhizosphere of wheat. Plants were grown in Chromosol and Vertosol under elevated CO_2_ (eCO_2_, 800 ppm) and ambient CO_2_ (aCO_2_, 400 ppm) for 10 weeks. ns, *, ** and *** indicate significance of two-sample Wilcoxon Mann-Whitney tests at *p* > 0.05, *p* < 0.05, *p* < 0.01 and *p* < 0.001, respectively.

**Figure S5** Scatterplots of mineralized phytate (µg g^-1^) to species richness, Shannon indices and Pielou evenness in the rhizosphere compartments of 0 mm, 1.5 mm and 3 mm, and the bulk soil of Chromosol and Vertosol.


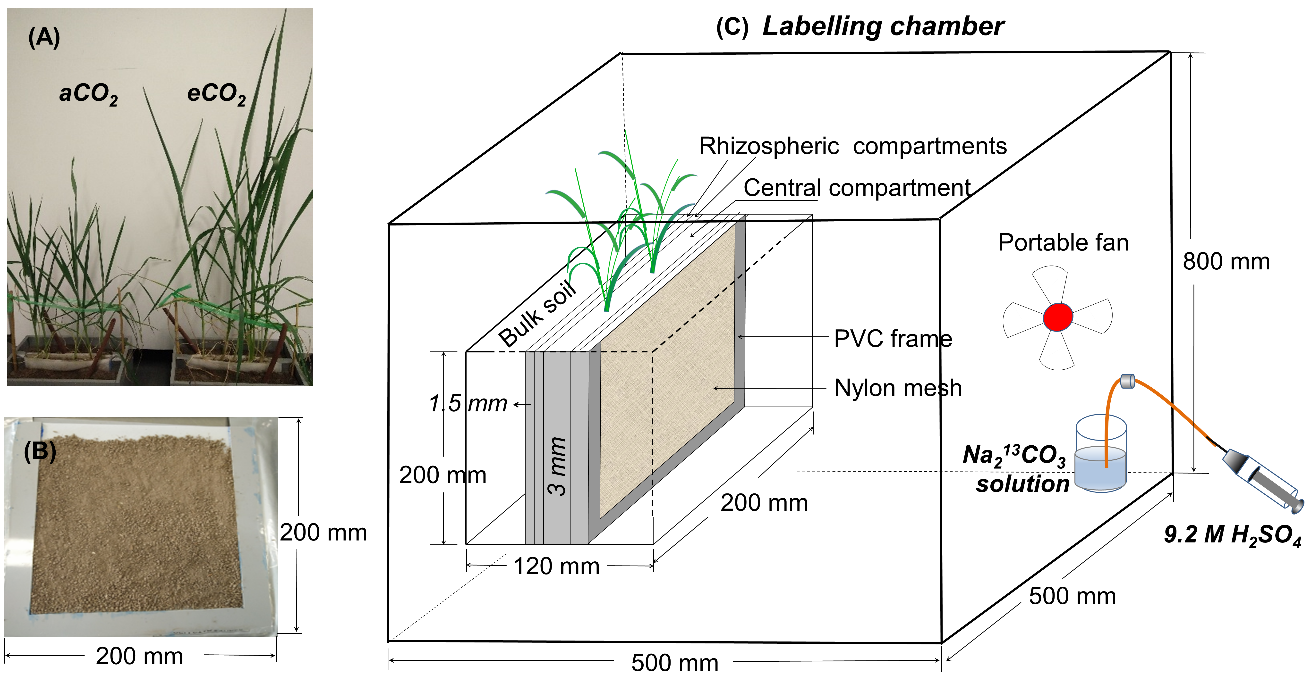


**Figure S6** The photos showing (A) plants grown for 10 weeks under ambient CO_2_ (aCO_2_, 400 ppm) and elevated CO_2_ (eCO_2_, 800 ppm), (B) a rhizo-compartment, and (C) a schematic structural diagram of the rhizobox in the ^13^C-labelling device used in the experiment.

**Table S1** The concentrations of total organic C, total P and Olsen P, soil pH and soil particle composition (texture) of the Chromosol and Vertosol used in the study

| Soil type | Total C  (g kg^-1^) | Total P  (mg kg^-1^) | Olsen P  (mg kg^-1^) | pH  (1:5 H_2_O) | Sand  (%) | Silt  (%) | Clay  (%) |
| --- | --- | --- | --- | --- | --- | --- | --- |
| Chromosol | 43 | 200 | 24.5 | 5.1 | 19 | 63 | 18 |
| Vertosol | 12 | 172 | 15.0 | 7.0 | 14 | 44 | 42 |
